# Investigation of the efficacy of fluralaner spot-on (Bravecto®) against infestations of *Ixodes holocyclus* on cats

**DOI:** 10.1186/s13071-018-2924-3

**Published:** 2018-06-26

**Authors:** Petr Fisara, Frank Guerino, Fangshi Sun

**Affiliations:** 1 MSD Animal Health, 26 Talavera Road, Macquarie Park, NSW Australia; 2 Merck Animal Health, 2 Giralda Farms, Madison, NJ 07940, USA

**Keywords:** Cat, Fluralaner, Spot-on, Tick, Paralysis, Australia, *Ixodes holocyclus*

## Abstract

**Background:**

Infestation of cats with the Australian paralysis tick, *Ixodes holocyclus* continues to be a threat because of the paralysis that can result from attachment of just a single tick. The outcome can be fatal, particularly if tick removal and treatment is not initiated soon after the onset of paralysis. However, there are no published studies to guide veterinarians and owners on preventive measures. A study was therefore initiated to determine the efficacy of a systemically-acting, spot-on formulation of fluralaner (Bravecto^®^) for cats against existing *I. holocyclus* infestations, and to investigate the duration of protection following a single administration.

**Methods:**

Healthy domestic cats, short or long-hair, immunized against holocyclotoxin, were randomly allocated to two groups of 10 cats per group, to receive either a single topically applied fluralaner treatment or no treatment. Fluralaner treatments were administered on Day 0 at a dose rate of 40 mg/kg. All cats were infested with 10 adult unfed female *I. holocyclus* on Day -1 and on Days 14, 28, 42, 56, 70 and 84. Ticks were assessed at 24 and 48 h after fluralaner treatment and 24, 48 and 72 hours after each subsequent re-infestation. Ticks were counted but not removed at the 24- and 48-h post-challenge assessments and were removed following the 72-h counts.

**Results:**

The efficacy of fluralaner spot-on against an existing *I. holocyclus* infestation was 100% at 48 h post treatment. Following re-infestations, efficacy remained at 100% at the 72-h assessments for all challenges from Day 14 to Day 84. Differences between mean live tick counts on treated *versus* control cats were significant at all time points from the first post-treatment assessment (*t*-test: *t*_(18)_ = 23.162; *P* < 0.0001) through the final challenge on Day 84 (*t*-test: *t*_(18)_ = 21.153; *P* < 0.0001). No treatment-related adverse events were observed and there were no abnormal observations at the product application sites.

**Conclusions:**

A single treatment of fluralaner spot-on was well tolerated and provided 100% efficacy against *I. holocyclus* ticks for at least 84 days. Fluralaner spot-on can be a valuable tool to prevent tick infestation in cats, and to control the risk of *I. holocyclus*-induced paralysis.

## Background

Infestations with the tick *Ixodes holocyclus* continue to be a major concern as a cause of paralysis and death in companion animals along the eastern coast of Australia, with thousands of cases reported in dogs and cats each year [1–3]. Areas with broadleaved tree coverage, temperate climate and high humidity, along with the presence of suitable wildlife hosts such as bandicoots provide the optimal climatic, landscape and wildlife-host characteristics that favour tick survival and proliferation [3–6]. Thus, dogs and cats along the Eastern seaboard of Australia from north eastern Victoria to northern Queensland are at greatest risk of *I. holocyclus* infestations, with increased threat in coastal areas of New South Wales and Queensland [3, 7, 8].

The paralysis that can result from an infestation with just a single *I. holocyclus* female is due to the tick’s production of neurotoxins that interfere with presynaptic release of acetylcholine in the affected host [9]. These neurotoxins (holocyclotoxins) are released in increasing quantities starting at approximately three days after *I. holocyclus* attaches, corresponding with a marked increase in the size and activity of the tick’s salivary glands [10, 11]. Clinical signs in affected hosts then begin to develop from three days after ticks begin feeding, with a potentially fatal paralysis progressing from four or five days after attachment [4, 10, 12, 13].

Reports of tick paralysis in cats have not been as thoroughly documented as in dogs, even though in the above-mentioned areas of Australia the condition is frequently diagnosed by veterinarians. A recent paper described a retrospective investigation of tick-induced paralysis in cats that were presented to four emergency clinics in Queensland between 2008 and 2016 [14]. A total of 2077 cats were diagnosed with tick paralysis over this period, and 273 of those cats either died or were euthanised mainly due to the cost of treatment, perhaps a testament to the significant expense of treating tick paralysis cases. Another survey found that 808 cases of tick paralysis in cats were reported from 96 clinics in Australia in a period of 17 months, between September 2010 and December 2012 [7]. The aim of another study was to describe the association between landscape and climate factors with the occurrence of tick paralysis cases in dogs and cats reported by veterinarians in Australia [3]. During the study period (October 2010 - December 2012), a total of 5560 cases [4235 (76%) canine; 1325 (24%) feline] were reported from 341 postcodes, mostly along the eastern seaboard of Australia and from the states of New South Wales and Queensland. These findings, while including only those cats that were presented for treatment, draw focus to the need for an effective tick control measure that can substantially reduce the risk of infestation, and therefore of the paralysis that may result.

To control tick infestations, cat owners in Australia have long been dependent upon repeated applications of topically applied products. The longest acting of these, a spray-on formulation of fipronil, needs to be administered every three weeks and relies on the entire body of the animal being covered, which in practice can be difficult to achieve on cats.

In dogs, a single oral administration of the novel long-acting isoxazoline fluralaner provided 100% efficacy against *I. holocyclus* infestations by 48 h post-treatment [15]. Protection against subsequent infestations extended through at least 112 days following a single treatment. However, for many owners of cats, oral formulations can be difficult to administer, and the topical route provides a more convenient and acceptable alternative. Therefore, a low-volume topical formulation of fluralaner (28% w/v) was developed, offering the potential to provide a high level of tick and flea control in cats.

Following application of this formulation to cats, fluralaner is readily absorbed, achieving maximum plasma concentrations between 3 and 21 days after treatment, with quantifiable plasma concentrations maintained for more than 12 weeks post administration [16]. These pharmacokinetic properties in cats suggest the potential to provide a quick kill of infesting *I. holocyclus* and to prevent tick paralysis over an extended period. Moreover, by acting systemically, this fluralaner spot-on product addresses a concern that traditional topically applied products, which kill parasites by contact, do not reliably reach some anatomic sites of tick attachment. These sites include the external anus, inside the anus, and among the transverse palatine ridges of the hard palate [4, 5]. A study was initiated to determine the efficacy of the fluralaner spot-on formulation against existing *I. holocyclus* infestations, and to investigate the duration of efficacy in controlling challenges throughout the 12 weeks following a single treatment.

## Methods

This was a randomized, negative-controlled unblinded study conducted at a veterinary laboratory in New South Wales, Australia. The study was conducted in compliance with the VICH Guidelines for Good Clinical Practice, VICH GL9, June 2000, the Australian Code for the Care and Use of Animals for Scientific Purposes, the Australian Pesticides and Veterinary Medicine Authority (APVMA) Guidelines for small animal ectoparasiticide efficacy submission, the APVMA Preamble for the World Association for Veterinary Parasitology Guidelines (WAAVP) guideline for fleas and ticks on dogs and cats, and the WAAVP guidelines for evaluating the efficacy of parasiticides for the treatment, prevention and control of flea and tick infestations on dogs and cats [17–21].

### Animals and housing

Cats selected for the study were sourced from the facility’s colony. The animals were required to be healthy, neutered or intact, able to handle the study procedures and to have been immunized to holocyclotoxin. The immunization was completed prior to the study commencement by gradual exposure of cats to unfed adult female *I. holocyclus*. During this period the cats were observed several times over a number of days after the last attachment of ticks. At the end of the process immunity was confirmed with a tick carrying capacity test.

From Day -8 to the end of the study, the cats were individually housed in pens with a floor area 1.5 × 3 m, equal areas located inside and outside, that allowed each cat to see neighbouring cats through a transparent door. Clean water was available *ad libitum* from water dispensers. All cats survived the study and returned to their original colony after the study completion.

### Inclusion/exclusion criteria

For inclusion in the study cats were required to be at least 14 weeks of age (actual age range one to 11 years), clinically normal and to have been shown to tolerate a Day -8 challenge with 10 *I. holocyclus* ticks for a duration of three days, while retaining at least 50% of the challenge without serious adverse effect. The infestation size of 10 ticks per cat was selected as the minimum number of ticks sufficient to allow a reliable statistical analysis. The cats could not have received either insecticidal or acaricidal treatments for at least 90 days prior to the commencement of the study. There was no restriction on body weight, and study cats weighed 2.8 to 7.2 kg.

### Randomization and treatment

Twenty-two cats were blocked by coat length (long or short) and within each block ranked by *I. holocyclus* counts on Day -5 (from the Day -8 infestation), and within that grouping ranked by ascending order of cat name. Two cats developed a local infection at the tick attachment site and were excluded from the study. From the 20 remaining cats, the two long-haired cats with the highest tick count formed Replicate 1; the next two formed Replicate 2. Replicate 3 consisted of the remaining long-haired cat with the lowest tick count and a short-haired cat with the lowest tick count. Replicates 4 to 10 included the remaining short-haired cats allocated from lowest to highest tick count. Within replicates each cat was randomly assigned to one of two treatment groups until there were 10 cats in each group. Cats were randomly assigned to individual pens within each pen block.

One group of cats served as untreated controls and cats in the other group were treated on Day 0 with fluralaner spot-on (Bravecto^®^, Merck Animal Health) at the proposed minimum fluralaner label dose rate of 40 mg/kg, using body weights recorded on Day -1.

The spot-on treatment was applied using a 1 ml plastic syringe to the dorsal midline at the junction of the top of the neck and the base of the skull, parting the hair coat to facilitate dose delivery at skin level. One or two spots were applied to each cat depending on the dose volume, with a maximum of 0.9 ml per spot. Treated cats were restrained by the handler for approximately one min to allow the treatment to spread at skin level and to prevent cats from shaking, licking, rolling or rubbing.

Immediately after treatment, and at one and two hours, the cats were observed for any adverse reactions. Skin examination was also carried out at 48 h after treatment and at 72 h after each infestation.

### Tick infestations and assessments

Adult unfed female *I. holocyclus*, collected from six separate localities within the Northern Rivers region of NSW were kept in jars in an incubator. Ten ticks were manually attached to each cat on Days -8, -1, 14, 28, 42, 56, 70 and 84. The infestations were carried out predominantly on the head, shoulders and dorsal midline, to simulate the ticks’ natural predisposition for these areas and to prevent the cats from grooming ticks off.

Tick counts began with a search of the areas to which the ticks were attached at the time of infestation. This was followed by a whole-body search to locate any ticks that may have migrated away from the tick application site. For all study procedures the control cats were handled first to avoid any potential transfer of product from treated to untreated cats. Ticks were counted and assessed at 24 and 48 h post-treatment and at 24, 48 and 72 h after each post-treatment infestation. The 24-h post-treatment and 24- and 48-h post-infestation assessments were completed without removing the ticks. Following the 48 h post-treatment and the 72-h post-infestation assessments all ticks were removed and discarded. The total weekly count consisted of all live and dead ticks found on each cat at 24 and 48 h post-treatment and 24, 48 and 72 h post-infestations. The ticks were classified as shown in Table 1 [15]. The assessment of efficacy was based on the number of category 1, 2, 3 and 7 ticks found on each cat at 48 h post-treatment and at 72 h after each post-treatment tick infestation.

**Table 1:** Tick assessment categories used to determine fluralaner acaricidal effect^a^

Category	Viability	Attachment status	Acaricidal effect
1	Live	Free, unattached	Not demonstrated
2	Live	Attached, un-engorged	Not demonstrated
3	Live	Attached, engorging/engorged	Not demonstrated
4	Dead	Free, unattached	Demonstrated
5	Dead	Attached, un-engorged	Demonstrated
6	Dead	Attached, engorging	Demonstrated
7	Dead	Attached, engorged	Not demonstrated

^a^Adapted from Marchiondo et al. [21] and Fisara & Webster [15]

All cats were monitored closely at least three times every day during the study for any signs of onset of paralysis. Particular attention was paid to signs of incoordination, paresis, dyspnea, or hind limb paralysis associated with tick paralysis, and to any signs of respiratory compromise.

### Statistical methods

Separate analyses (control *vs* fluralaner spot-on) were conducted at each tick count time point. The logarithm of (count + 1) transformation was applied to all observations prior to analysis. The log transformation of tick counts (ticks in categories 1, 2, 3 and 7) 72 h after each infestation (including 48 h post-treatment) were analyzed using a linear mixed model including treatment group as a fixed effect. Least squares means were used for treatment comparisons. A Kenward-Rogers adjustment was used to determine the denominator degree of freedom for hypothesis. The null hypothesis was that there would be no significant differences among treatment groups within each count week. Statistical significance was declared when *P* ≤ 0.05. Two-tailed tests were used for the comparison among treatment groups. The primary software was SAS version 9.3.

## Results and discussion

**Fig. 1:**
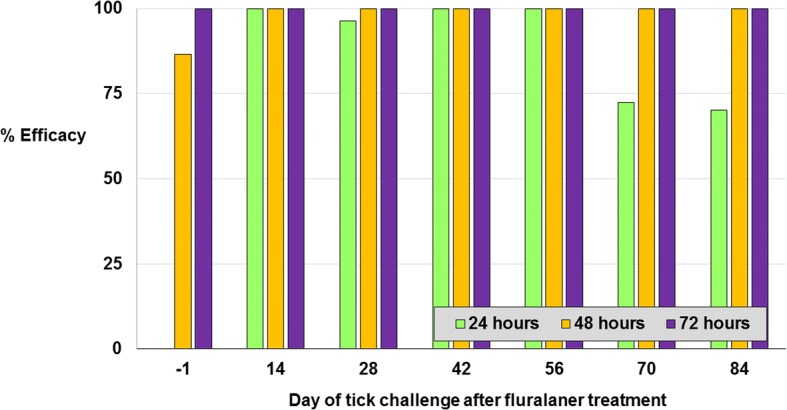
Topically administered fluralaner efficacy against *Ixodes holocyclus* at 24 or 48 h post-treatment and subsequently at 24, 48 and 72 h after challenge

The study methodology was successful in maintaining tick infestations in control cats with mean counts in the group ranging from 5.5 to 7.3 ticks (Table 2). At 48 h post-treatment, tick counts for the fluralaner-treated cats were significantly lower than those of the untreated cats (*t*-test: *t*_(18)_ = 23.162; *P* < 0.0001), with significant differences maintained at 72 h after subsequent challenges through the final tick assessment on Day 87 (*t*-test: *t*_(18)_ = 21.153; *P* < 0.0001). The efficacy of the fluralaner spot-on treatment was therefore 100% at 48 h post-treatment and remained at 100% at 48 and 72 h after all subsequent experimental infestations of *I. holocyclus* (Fig. 1). This is within the critical 72-h post-attachment period, before the risk of paralysis may begin to develop from the injection of neurotoxins that are produced in the tick salivary glands after three days of attachment [10, 11]. The results of this study parallel results of a similar study in dogs in which orally administered fluralaner provided efficacy of 100% against existing infestations with *I. holocyclus* within 48 h post-treatment, sustained through 72 h following challenges at two-weekly intervals through 84 days and at 112 days post-treatment [15].

**Table 2:** Mean live *Ixodes holocyclus* counts and efficacy of fluralaner applied once topically to cats^a^

	Day of infestation						
	Day -1	14	28	42	56	70	84
Untreated control							
Arithmetic mean	7.3	6.2	6.4	6.2	5.5	6.8	5.9
Geometric mean	7.0	5.8	5.6	5.8	4.6	6.5	5.7
Fluralaner spot-on							
Arithmetic mean	0.0	0.0	0.0	0.0	0.0	0.0	0.0
Efficacy (%)	100.0	100.0	100.0	100.0	100.0	100.0	100.0
Geometric mean	0.0	0.0	0.0	0.0	0.0	0.0	0.0
Efficacy (%)	100.0	100.0	100.0	100.0	100.0	100.0	100.0
Comparison	*t*_(18.0)_ = 23.2, *P* < 0.0001	*t*_(18.0)_ = 15.8, *P* < 0.0001	*t*_(18.0)_ = 10.7, *P* < 0.0001	*t*_(18.0)_ = 17.7, *P* < 0.0001	*t*_(18.0)_ = 8.9, *P* < 0.0001	*t*_(18.0)_ = 22.4, *P* < 0.0001	*t*_(18.0)_ = 21.1, *P* < 0.0001

^a^Efficacy was based on an assessment of tick viability at 48 h post-treatment and at 72 h post challenge infestations

Treatment was well tolerated, with no observations of adverse events, either systemic or at the site of product application. Abscesses caused by bacterial infections were observed at sites of tick attachment in a number of the untreated control group cats but not in fluralaner-treated cats. One control group cat was removed from the study on Day 62, due to an abscess between the shoulders that required surgical treatment and was replaced by an untreated cat. Fluralaner spot-on treatment was apparently effective at eliminating the risk of local abscess development secondary to *I. holocyclus* attachment.

Currently, the only product registered in Australia to provide control of *I. holocyclus* infestations on cats for more than three days is a topically applied pump-spray formulation of fipronil for application every 21 days. An average size cat (4 to 5 kg body weight) requires up to 20 pumps to cover the entire body. Therefore, treatment with this product presents a compliance challenge for the typical cat owner who must restrain the cat, pump the spray and effectively apply the treatment to the cat’s entire body. Moreover, there are no published data on the efficacy of this topical treatment for controlling *I. holocyclus* infestations.

Use of topically applied, but systemically distributed fluralaner for effective control of feline infestations with *I. holocyclus* can provide a more convenient option for owners wishing to protect their cats against paralysis tick infestations. It has been suggested that owner compliance for ectoparasite control measures for dogs should improve through the use of products that allow extended between-treatment intervals [22]. It is reasonable to assume the same result could occur for cat owners using this long-acting low volume spot-on formulation of fluralaner. An additional advantage of this systemically-acting formulation over the fipronil product is that efficacy would not be affected when ticks attach at extremities, such as the anus and between the toes, where topical products acting by contact do not reliably achieve acaricidal concentrations.

To our knowledge, this report is the first to describe the efficacy of any treatment to control *I. holocyclus* infestations in cats. The results demonstrate that treatment of cats with fluralaner can provide sustained protection from infestations with *I. holocyclus*, for up to 84 days after treatment. The availability of a convenient spot-on product with high and sustained efficacy offers a breakthrough in preventing holocyclotoxin-induced paralysis in cats.

## Conclusions

Treatment of cats with fluralaner spot-on provides 100% efficacy against *I. holocyclus* challenge for at least 84 days. There were no treatment-related adverse events and no abnormal observations at the product application site. While inherent variations between animals, animal owners and tick challenges preclude any claim for any product of 100% efficacy on every occasion, the results of our study provide substantial reassurance that application of this fluralaner spot-on can control the risk of establishment of *I. holocyclus* infestations and can prevent the development of tick paralysis in cats.

## Abbreviations

APVMA: Australian Pesticides and Veterinary Medicine Authority
VICH: International Cooperation on Harmonisation of Technical Requirements for Registration of Veterinary Medicinal Products
WAAVP: World Association for Veterinary Parasitology Guidelines
